# Evaluation of electrocardiographic parameters in patients with hearing loss genotyped for the connexin 26 gene (GJB2) mutations^☆^

**DOI:** 10.1016/j.bjorl.2016.02.008

**Published:** 2016-04-22

**Authors:** Agnieszka Sanecka, Elzbieta Katarzyna Biernacka, Magdalena Sosna, Malgorzata Mueller-Malesinska, Rafal Ploski, Henryk Skarzynski, Ryszard Piotrowicz

**Affiliations:** a Institute of Cardiology, Warsaw, Poland; b Institute of Physiology and Pathology of Hearing, World Hearing Center, Nadarzyn, Poland; c Medical University of Warsaw, Department of Medical Genetics, Warsaw, Poland

**Keywords:** ECG, Hearing loss, GJB2 mutation, Connexin 26, ECG, Perda auditiva, Mutação de GJB2, Conexina 26

## Abstract

**Introduction:**

Several studies have associated congenital sensorineural hearing loss in children with prolongation of the cardiac parameter QTc. The cause of this association is unknown. At the same time, mutations in GJB2, which encodes connexin 26, are the most common cause of congenital hearing impairment.

**Objective:**

To compare electrocardiographic parameters (PR interval, QRS complex, and QTc interval) in patients with hearing loss who were tested for mutations in GJB2 and GJB6 to investigate whether these mutations affect electrical activity of the heart.

**Methods:**

346 patients (176 males, 170 females) with sensorineural hearing loss of 30 dB HL or more, aged 21.8 ± 19.9 years (including 147 children <14 years), underwent both genetic study for GJB2 and GJB6 mutations and electrocardiography.

**Results:**

Mutations in GJB2, including homozygotes and heterozygotes, were found in 112 (32%) patients. There were no significant differences in ECG parameters between groups of patients with and without mutations in GJB2. No differences were observed either in men (mean PR with mutation: 155 ± 16.6 vs. 153.6 ± 30.1 without; QRS: 99.9 ± 9.9 vs. 101.1 ± 15.4; QTc: 414.9 ± 29.9 vs. 412.4 ± 25.7) or women (mean PR with: 148.7 ± 21 vs. 143.8 ± 22.8 without; QRS: 94.8 ± 7.6 vs. 92.9 ± 9.6; QTc: 416.8 ± 20.6 vs. 424.9 ± 22.8). In similar fashion, we did we find any significant differences between groups of children with and without GJB2 mutations (mean PR with: 126.3 ± 19.6 vs. 127 ± 19.7 without; QRS: 80.7 ± 9.5 vs. 79.4 ± 11.6; QTc: 419.7 ± 23.5 vs. 419.8 ± 24.8).

**Conclusion:**

No association was found between the presence of GJB2 mutations encoding connexin 26 in patients with hearing loss and their ECG parameters (PR, QRS, QTc).

## Resumo

**Introdução:**

Vários estudos têm associado a perda auditiva neurossensorial congênita em crianças ao prolongamento do parâmetro cardíaco QTc. A causa desta associação é desconhecida. Ao mesmo tempo, as mutações no GJB2, que codifica a conexina 26, são a causa mais comum de deficiência auditiva congênita.

**Objetivo:**

Comparar parâmetros eletrocardiográficos (intervalo PR, complexos QRS e intervalo QTc) em pacientes com perda auditiva que foram testados para mutações no GJB2 e GJB6 para investigar se estas mutações afetam a atividade elétrica do coração.

**Método:**

Ao todo, 346 pacientes (176 homens, 170 mulheres) com perda auditiva neurossensorial de 30 dB ou mais, com média de idade de 21,8 ± 19,9 anos (incluindo 147 crianças <14 anos), foram submetidos a estudo genético para mutações de GJB2 e GJB6 e eletrocardiograma.

**Resultados:**

Mutações no GJB2, incluindo homozigóticos e heterozigóticos, foram encontradas em 112 (32%) pacientes. Não houve diferenças significativas nos parâmetros de ECG entre grupos de pacientes com e sem mutações no GJB2. Não foram observadas diferenças em homens (PR médio com mutação: 155 ± 16,6 vs 153,6 ± 30,1 sem mutação; QRS: 99,9 ± 9,9 vs 101,1 ± 15,4; QTc: 414,9 ± 29,9 vs 412,4 ± 25,7) nem em mulheres (PR médio com: 148,7 ± 21 vs. 143,8 ± 22,8, sem; QRS: 94,8 ± 7,6 vs 92,9 ± 9,6; QTc: 416,8 ± 20,6 vs 424,9 ± 22,8). Da mesma forma, encontramos diferenças significativas entre os grupos de crianças com e sem mutações de GJB2 (PR médio com: 126,3 ± 19,6 vs 127 ± 19,7, sem; QRS: 80,7 ± 9,5 vs 79,4 ± 11,6; QTc: 419,7 ± 23,5 vs. 419,8 ± 24,8).

**Conclusão:**

Não foi encontrada associação entre a presença de mutações de GJB2 que codificam conexina 26 em pacientes com perda auditiva e seus parâmetros de ECG (PR, QRS, QTc).

## Introduction

Connexins are integral transmembrane proteins which form channels in the cell membrane called connexons. In adjacent cells, connexons form tight junctions that are necessary for transfer of electrical signals and water-soluble molecules. Connexons help maintain tissue homeostasis and assist in the management and regulation of a cell's ionic environment.^1^

Connexins are present throughout the human body. Even though cells can contain a number of different connexins, each plays a separate role. In humans, there are 21 proteins belonging to the connexin family, and each is encoded by a different gene.^2^ Mutations in genes encoding connexins create disturbances in their structure and function, which can lead to hereditary diseases such as deafness, cataracts, skin diseases, and neurodegenerative conditions.^3^, ^4^, ^5^, ^6^

In the cells of the inner ear, connexin 26 and connexin 30 are involved in potassium ion transport processes necessary for hearing. When a hair cell is stimulated, potassium ions in the endolymph enter the cilia and body of the cell, causing it to depolarize. Due to gap-junction connections, ions are in turn transported by supporting cells and cells in stria vascularis to endolymph. Via this recirculation of potassium ions, a highly positive (+80 mV) endolymphatic potential is generated, a necessary condition for hearing. Disruption of this mechanism causes potassium ion intoxication and tissue damage.^7^

Both the GJB2 (MIM*121011) gene (encoding connexin 26) and the GJB6 (MIM*604418) gene (encoding connexin 30) are located on chromosome 13 (13q11–q12). Mutations of the GJB2 gene lead to disturbances in the functioning of connexin 26, causing hearing loss (HL) by impairing transfer of intracellular potassium ions within the inner ear.^8^ GJB2 mutations are a major cause of autosomal recessive nonsyndromic HL in many populations. Among Europeans, the most common pathogenic mutation in the GJB2 gene is a deletion of guanine at position 35 (NM_004004.5:c.35delG).^9^, ^10^ In most cases (70%), HL occurs as an isolated defect; in other instances it is accompanied by heart, craniofacial, kidney, skin, and other defects.

Several studies have reported an association of congenital sensorineural HL with prolongation of QTc interval in children. The cause of this association is unknown.^11^, ^12^

This study measured electrocardiographic parameters in patients with HL, who were also tested for genetic mutation. Analysis of the electrocardiogram (ECG) provided PR interval, QRS complex, and QTc interval, and these electrocardiographic parameters were compared in two groups of patients: one with HL caused by mutations in the GJB2 gene and another with HL and no mutation in this gene. In this way, it was possible to investigate whether there was a relationship between connexin 26 and connexin 30 functions and cardiac electrical activity.

## Methods

### The study population

The study group consisted of 346 consecutive patients, aged 21.8 ± 19.9 years (0.16–84 years), including 176 males and 170 females, with sensorineural HL of 30 dB or more (calculated as the average hearing threshold at frequencies relevant for speech – 0.5, 1, 2, and 4 kHz) and on whom genetic tests were done to identify mutations in GJB2 and GJB6. The HL was confirmed by pure tone audiometry or, in the case of children less than 6 years old, Brainstem Evoked Response Audiometry (BERA). Among the subjects studied, 91 had cochlear implants.

### Genetic tests

DNA was extracted from the peripheral blood by a salting-out procedure.^13^ The typing for the c.35delG mutation in GJB2 gene was performed by two independent methods: AS-PCR and PCR-RFLP, as described previously.^14^, ^15^ Briefly, multiplex amplification of three GJB2 regions (c.12-72, c.68-198 and c.306-464) with fluorescent labeled primers was performed. In parallel, PCR-RFLP analysis specific for c.35delG mutation was conducted. Size of the fragments generated by the two above-mentioned protocols was determined using a 3500XL Genetic Analyzer (Life Technologies) with Gene Mapper (Life Technologies) software. Samples lacking two c.35delG mutations were further analyzed by direct DNA sequencing of the PCR amplified exon 2 of the gene. Sequence analysis was performed with Variant Reporter v1.1 (Life Technologies) software. Genotyping of the c.-23+1G>A GJB2 mutation was performed using real-time PCR according to the manufacturer's instructions (Life Technologies), while the GJB6 mutation del (GJB6-D13S1830) was detected using the method of del Castillo et al.^16^ Genetic nomenclature is in line with recommendations of the Human Genome Variation Society (HGVS).

### Electrocardiographic tests

All 346 patients underwent a 12 lead Electrocardiogram (ECG). ECG recording was performed and evaluated using the Sentinel Cardiology Information Management System (Reynolds Medical). The durations of PR interval, QRS complex, and QTc interval were measured by a single observer on a 5 times enlarged image on a computer monitor with a cursor accuracy of 1 ms (sampling frequency 1000 Hz). PR interval was usually measured in lead II, QRS complex in V1 and/or V5, and QT interval in at least three leads of the same cycle (usually leads II, V2, and V5). Heart rate was calculated from the RR interval preceding the cycle of the QT interval measured. To correct QT interval due to heart rate (i.e., QTc), Bazett's formula was used for rates between 50 and 120 bpm; for faster or slower rates Hodges’ formula was used. In order to calculate average QTc, patients with QRS complexes ≥120 ms were excluded. A QTc interval above 460 ms in women, 450 ms in men, and 440 ms in children under 14 years of age, was diagnosed as prolonged.

The study population was divided into three groups based on reference values of QTc for children, women, and men,^17^ giving 147 children <14 years (42.4% of patients), 100 women ≥14 years (28.9%), and 99 men ≥14 years (28.6%). The group of children included 70 (47%) girls.

### Statistical analysis

Statistical analysis of each of the three groups involved a Kruskal–Wallis test and a Wilcoxon two-sample test.

### Other

The study was approved by the Bioethics Committee, approval number KB/05/2009. The study was done in accordance with the principles of the Declaration of Helsinki. All patients or their guardians gave written consent to participate.

## Results

### Characteristics of the study population and genetic studies

Mutations in GJB2 were identified in 112 (32%) patients with HL. No mutations were detected in the GJB2 and GJB6 genes analyzed in the other 234 (68%).

The mean age of children with a GJB2 mutation and children without GJB2 mutation was similar (6.0 ± 4.6 vs. 5.8 ± 4.5 years, *p* = 0.86). The mean age of adults in both groups (with and without GJB2 mutation) was also similar (35.1 ± 17.6 vs. 30.5 ± 16.5 years, *p* = 0.17 for women; 31.6 ± 18.9 and 30.3 ± 17.5 years, *p* = 0.99 for men) (Table 1).

**Table 1:** Characteristics of the group of patients diagnosed with mutations in gene GJB2 (genotype homozygous and heterozygous together) and the group of patients without any mutation in the gene.

	Group of patients with GJB2 mutation	Group of patients without GJB2 mutation
Number of patients – total	112	234
Number of children <14 years (% of group)	52 (46.4%)	95 (40.6%)
Number of women ≥14 years	34	66
Number of men ≥14 years	26	73
Mean age (years) in performance of ECG	20.8	20.2
Number (%) of patients in the group after the implantation of a screw	28 (25%)	62 (26%)
Number (%) of patients with a significant HL	55 (48%)	103 (44%)
Number (%) of patients with an average degree of HL	6 (5%)	30 (8%)
Number (%) of patients with congenital HL (*n* = 200)	62 (55%)	138 (59%)

The homozygous genotype c.[35delG];[(35delG)] in the GJB2 gene was detected in 72 (20.8%) subjects. In 40 (11.8%) individuals, heterozygous GJB2 genotypes were identified: c.[35delG];[=] (*n* = 15), c.[35delG(;)313_326del] (*n* = 5), c.[35delG(;)-23+1G>A] (*n* = 4), c.[313_326del];[=] (*n* = 2), c.[35delG(;)551G>C] (*n* = 2), c.[35delG(;)95G>A] (*n* = 2), and a compound GJB2 and GJB6 heterozygous genotype [GJB2:c.35delG];[GJB6:del(GJB6-D13S1830)] (*n* = 2). The following GJB2 heterozygous genotypes occurred in individual cases: c.[35delG(;)269T>C], c.[148G>A];[=], c.[79G>A; 341G>A; 269T>C], c.[101T>C];[=], c.[35delG(;)235delC], c.[35delG(;)358_360delGAG], c.[35delG(;)139G>T], c.[334_335delAA];[=] (Table 2).

**Table 2:** Types of genotype and their distribution in the study population.

Genotype GJB2	Children		Adults	
	Girls	Boys	Women	Men
c.[35delG];[(35delG)]	15	19	21	17
[GJB2:c.35delG];[GJB6:del(GJB6-D13S1830)]	1	1		
c.[35delG(;)313_326del]	2	1	1	1
c.[35delG(;)-23+1G>A]		1	2	1
c.[35delG];[=]	4	3	6	2
c.[35delG(;)269T>C]	1			
c.[35delG(;)235delC]			1	
c.[35delG(;)358_360delGAG]				1
c.[35delG(;)551G>C]			1	1
c.[35delG(;)95G>A]	1		1	
c.[35delG(;)139G>T]				1
c.[148G>A];[=]		1		
c.[313_326del];[=]		1	1	
c.[79G>A; 341G>A; 269T>C]				1
c.[334_335delAA];[=]				1
c.[101T>C];[=]		1		

### Electrocardiographic findings

The next phase of the study was to compare electrocardiographic parameters in the study population. The measured parameters were compared between two groups: the group of patients with a mutation in gene GJB2 (both homozygotes and heterozygotes) and the group of patients with no mutation in the gene. No significant differences were found for any measured parameter in any of the groups studied. Table 3 presents inter-group differences in PR interval; Table 4 presents inter-group differences in QRS complex; and Table 5 presents inter-group differences in QTc interval.

**Table 3:** Findings of PR interval in the ECG study population.

Group^a^	Patients with a mutation in GJB2			Patients without any mutation in GJB2			*p* (NS)
	Mean PR ± SD (ms)	Lowest value (ms)	Highest value (ms)	Mean PR ± SD (ms)	Lowest value (ms)	Highest value (ms)	
Children (*n* = 44/78)	126.3 ± 19.6	103	186	127 ± 19.7	100	177	0.74
Women (*n* = 33/57)	148.7 ± 21.0	111	192	143.8 ± 22.8	111	264	0.11
Men (*n* = 23/57)	155.0 ± 16.6	114	176	153.6 ± 30.1	116	265	0.12

a Numbers in brackets indicate the number of patients with/without GJB2 mutation.

**Table 4:** Findings of QRS complex in the ECG study population.

Group	Patients with a mutation in GJB2			Patients without any mutation in GJB2			*p* (NS)
	Mean QRS ± SD (ms)	Lowest value (ms)	Highest value (ms)	Mean QRS ± SD (ms)	Lowest value (ms)	Highest value (ms)	
Children (*n* = 49/89)^a^	80.7 ± 9.5	65	99	79.4 ± 11.6	61	114	0.32
Women (*n* = 33/59)	94.8 ± 7.6	80	110	92.9 ± 9.6	65	118	0.31
Men (*n* = 23/59)	99.9 ± 9.9	85	119	101.1 ± 15.4	65	156	0.98

a Numbers in brackets indicate the number of patients with/without GJB2 mutation.

**Table 5:** Findings of QTc interval in the ECG study population.

Group^a^	Patients with a mutation in GJB2			Patients without any mutation in GJB2			*p* (NS)
	Mean QTc ± SD (ms)	Lowest value (ms)	Highest value (ms)	Mean QTc ± SD (ms)	Lowest value (ms)	Highest value (ms)	
Children (52/94)	419.7 ± 23.5	366	479	419.8 ± 24.8	371	483	0.91
Women (34/64)	416.8 ± 20.6	371	456	424.9 ± 22.8	370	467	0.08
Men (26/61)	414.9 ± 29.9	366	485	412.4 ± 25.7	362	476	0.86

a Numbers in brackets indicate the number of patients with/without GJB2 mutation.

In addition, we compared parameters in patients with profound HL caused by a GJB2 mutation homozygous genotype c.[35delG];[(35delG)] and in those with a heterozygous genotype [GJB2:c.35delG];[GJB6:del(GJB6-D13S1830)] versus all other patients with other types of HL. The analysis revealed no significant differences (Table 6).

**Table 6:** Findings of PR interval, QRS complex, and QTc interval in 2 groups. Group A, patients with homozygous genotype c.[35delG];[(35delG)] and heterozygous genotype [GJB2:c.35delG];[GJB6:del(GJB6-D13S1830)]; group B, all other patients (with other heterozygous genotype and without any mutation in GJB2).

	Children (*n* = 31/90)^a^	Women (*n* = 20/70)	Men (*n* = 15/58)
Mean PR ± SD (ms) in group A	122.55 ± 18.51	144.1 ± 22.98	153.8 ± 15.07
Mean PR ± SD (ms) in group B	128.02 ± 25.38	146.3 ± 25.74	154.05 ± 25.55
*p*	0.0633	0.3934	0.1174

	Children (*n* = 33/104)	Women (*n* = 20/72)	Men (*n* = 15/60)
Mean QRS ± SD (ms) in group A	79.81 ± 9.81	94.05 ± 7.91	98.6 ± 10.18
Mean QRS ± SD (ms) in group B	79.87 ± 13.2	93.24 ± 13.17	101.27 ± 13.88
*p*	0.4528	0.4124	0.4577

	Children (*n* = 36/111)	Women (*n* = 20/79)	Men (*n* = 17/74)
Mean QTc ± SD (ms) in group A	418.33 ± 20.4	417.3 ± 18.45	412.41 ± 32.65
Mean QTc ± SD (ms) in group B	420.5 ± 24.16	423.74 ± 24.07	414.47 ± 24.16
*p*	0.4462	0.0908	0.4594

a Numbers in brackets indicate the number of patients in group A/B, respectively.

Finally, we analyzed the incidence of ECG abnormalities in the group of patients with a mutation in GJB2 and those without any mutation in this gene. The abnormalities in the group of patients with a mutation were: atrial rhythm (2 patients), sinus bradycardia (*n* = 12), short time atrioventricular conduction (*n* = 3), left anterior fascicular block (*n* = 1), a QTc interval above the norm for age and sex (*n* = 10), and signs of myocardial inferolateral wall necrosis associated with prolonged QTc (*n* = 1). In the group of patients without any mutation, abnormalities were: sinus bradycardia (*n* = 25), atrial rhythm (*n* = 3), wandering atrial pacemaker (*n* = 1), short time atrioventricular conduction (*n* = 2), first degree atrioventricular block (*n* = 5), incomplete right bundle branch block (*n* = 1), left anterior fascicular block (*n* = 1), complete bundle branch block (*n* = 7), intraventricular conduction disturbances (*n* = 3), signs of left ventricular hypertrophy (*n* = 3), signs of myocardial ischemia (*n* = 2), and prolonged QTc (*n* = 23). In one boy, there was supraventricular rhythm with a prolonged atrioventricular conduction time, nonspecific intraventricular conduction disturbances, and signs of right ventricular hypertrophy.

In the group of patients with a GJB2 mutation and prolonged QTc (*n* = 10), the mean QTc interval was 463.2 ± 15.6 ms, whereas in patients with prolonged QTc and without any mutation (*n* = 23), the mean QTc interval was 460.3 ± 12.8 ms. This difference was also not significant (*p =* 0.69).

## Discussion

Our study acquired ECGs from 346 patients with HL who were tested for mutations in GJB2 and GJB6 genes. There is a general lack of data on detailed ECG parameters in similarly large populations. There have been reports of normal ECGs performed on 11 patients aged 9–20 years in 5 families with HL associated with GJB2 mutations.^18^ In addition, another study^19^ described 270 children with sensorineural HL of which 143 underwent an ECG: abnormalities were detected in 10 (7%), 3 had prolonged QT interval (1 had multiple atrial and ventricular septal defects and 2 were both diagnosed with Jervell and Lange–Nielsen syndrome), and other reported abnormalities included sinus bradycardia, right bundle branch block, and left ventricular hypertrophy. Of 220 children in the study group, 33 (15%) had mutations in both alleles of the GJB2 gene.

Our study is the first to our knowledge to describe ECG parameters such as PR interval and QRS complex in a large group of patients with HL. GJB2 gene mutations cause HL in 20–60% of Caucasian children. The prevalence of GJB2 mutations in the European population is highest in Mediterranean countries and lowest in the north of the continent. It is estimated that the c.35delG mutation is carried by 1–3% of the European population.^20^, ^21^ The most common GJB2 genotype occurring in European patients with HL is the homozygous genotype c.[35delG];[(35delG)].^20^, ^21^ In our study group of patients with HL, this genotype was also the most common. Furthermore, we have shown that among the population studied there were also other mutations, although they were rare. One rare mutation, the c.167delT reported by Dong et al.^22^ should be noted, however, as this mutation is uncommon in Europe generally but much more frequent in Ashkenazi Jews.^23^ In contrast, the most common mutation in Japanese is c.235delC, where its prevalence is 1–2.1%.^24^

In addition to connexin 26, a similar biological role in the inner ear is also contributed by connexin 30, encoded by the GJB6 gene, whose locus is located in the immediate vicinity of the GJB2 gene on chromosome 13.^25^, ^26^ Deletion of 342 kb encompassing the GJB6 gene has been described, particularly in Spanish patients. Further studies have shown that the phenotypic effects of mutations in both genes are identical, and a compound heterozygous genotype [GJB2:c.35delG];[GJB6:del(GJB6-D13S1830)] is characterized by hearing loss.^16^

Our assessment of electrocardiographic parameters in patients with HL caused by GJB2 mutation has shown that atrioventricular conduction (defined by PR interval), intraventricular conduction time (defined by QRS duration), and the QTc interval did not differ significantly in comparison to patients with HL who did not have any mutations in this gene.

### Limitations of the study

Our study did contain some limitations. Different phenotypic expressions are the result of various GJB2 mutations. Since the described GJB2 gene mutations that result in HL are recessive, we compared parameters from a group of patients with a homozygous c.[35delG];[(35delG)] genotype and a heterozygous [GJB2:c.35delG];[GJB6:del(GJB6-D13S1830)] genotype with all other patients. The results of such an analysis are limited by the small number of people with a homozygous c.[35delG];[(35delG)] genotype, and there were only 2 children with heterozygous [GJB2:c.35delG];[GJB6:del(GJB6-D13S1830)] genotype giving the phenotypic effect of deafness.

In the study group, the presence of heart defects has not been ruled out by a reliable test (e.g. echocardiography), and neither has the effect of certain medications which might have influenced electrocardiographic parameters. The study group was selected by screening of people with HL who did not report symptoms of heart disease or did not take drugs, producing a group of apparently healthy people who had HL.

## Conclusion

In patients with HL, no association was found between ECG parameters and the presence of GJB2 mutations encoding connexin 26.

## Conflicts of interest

The authors declare no conflicts of interest.

## References

[1] Rutkowski R., Kosztyła-Hojna B., Kańczuga-Koda L., Sulkowska M., Sulkowski S., Rutkowski K. (2008). Structure and physiological function of connexin proteins. Postepy Hig Med Dosw.

[2] Willecke K., Eiberger J., Degen J., Eckardt D., Romualdi A., Güldenagel M. (2002). Structural and functional diversity of connexin genes in the mouse and human genome. Biol Chem.

[3] Richard G. (2003). Connexin gene pathology. Clin Exp Dermatol.

[4] Wei C.J., Xu X., Lo C.W. (2004). Connexins and cell signaling in development and disease. Annu Rev Cell Dev Biol.

[5] Zoidl G., Dermietzel R. (2010). Gap junctions in inherited human disease. Pflugers Arch.

[6] Kelsell D.P., Dunlop J., Stevens H.P., Lench N.J., Liang J.N., Parry G. (1997). Connexin 26 mutations in hereditary non-syndromic sensorineural deafness. Nature.

[7] Lefebevre P.P., Van de Water T.R. (2000). Connexins, hearing and deafness: clinical aspects of mutations in connexin 26 gene. Brain Res Rev.

[8] Snoeckx R.L., Huygen P.L., Feldmann D., Marlin S., Denoyelle F., Waligora J. (2005). GJB2 mutations and degree of hearing loss: a multicenter study. Am J Hum Genet.

[9] Mahdieh N., Rabbani B. (2009). Statistical study of 35delG mutation of GJB2 gene: a meta-analysis of carrier frequency. Int J Audiol.

[10] Wiszniewski W., Sobieszczanska-Radoszewska L., Nowakowska-Szyrwinska E., Obersztyn E., Bal J. (2001). High frequency of GJB2 gene mutations in Polish patients with prelingual nonsyndromic deafness. Genet Test.

[11] El Habbal M.H., Mahoney C.O. (2002). QT interval in children with sensory neural hearing loss. Pacing Clin Electrophysiol.

[12] Ilhan A., Tuncer C., Komsuoglu S.S., Kali S. (1999). Jervell and Lange–Nielsen syndrome: neurologic and cardiologic evaluation. Pediatr Neurol.

[13] Miller S.A., Dykes D.D., Polesky H.F. (1988). A simple salting out procedure for extracting DNA from human nucleated cells. Nucleic Acids Res.

[14] Pollak A., Skórka A., Mueller-Malesińska M., Waligóra J., Korniszewski L., Skarżyński H. (2005). Nowa metoda diagnostyki molekularnej niedosłuchu uwarunkowanego genetycznie. Audiofonologia.

[15] Storm K., Willocx S., Flothmann K., Van Camp G. (1999). Determination of the carrier frequency of the common GJB2 (connexin-26) 35delG mutation in the Belgian population using an easy and reliable screening method. Hum Mutat.

[16] del Castillo I., Villamar M., Moreno-Pelayo M.A., del Castillo F.J., Alvarez A., Tellería D. (2002). A deletion involving the connexin 30 gene in nonsyndromic hearing impairment. New Engl J Med.

[17] Baranowski R., Wojciechowski D., Maciejewska M. (2010). Zalecenia dotyczące stosowania rozpoznań elektrokardiograficznych. Dokument opracowany przez Grupę Roboczą powołaną przez Zarząd Sekcji Elektrokardiologii Nieinwazyjnej i Telemedycyny Polskiego Towarzystwa Kardiologicznego pod patronatem Polskiego Towarzystwa Kardiologicznego. Kardiol Pol.

[18] Cohn E.S., Kelley P.M., Fowler T.W., Gorga M.P., Lefkowitz D.M., Kuehn H.J. (1999). Clinical studies of families with hearing loss attributable to mutations in the connexin 26 gene (GJB2/DFNB1). Pediatrics.

[19] Lin J.W., Chowdhury N., Mody A., Tonini R., Emery C., Haymond J. (2011). Comprehensive diagnostic battery for evaluating sensorineural hearing loss in children. Otol Neurotol.

[20] Gasparini P., Rabionet R., Barbujani G., Melçhionda S., Petersen M., Brøndum-Nielsen K. (2000). High carrier frequency of the 35delG deafness mutation in European populations. Genetic Analysis Consortium of GJB2 35delG. Eur J Hum Genet.

[21] Lucotte G., Mercier G. (2001). Meta-analysis of GJB2 mutation 35delG frequencies in Europe. Genet Test.

[22] Dong J., Katz D.R., Eng C.M., Kornreich R., Desnick R.J. (2001). Nonradioactive detection of the common Connexin 26 167delT and 35delG mutations and frequencies among Ashkenazi Jews. Mol Genet Metab.

[23] Lerer I., Sagi M., Malamud E., Levi H., Raas-Rothschild A., Abeliovich D. (2000). Contribution of connexin 26 mutations to nonsyndromic deafness in Ashkenazi patients and the variable phenotypic effect of the mutation 167delT. Am J Med Genet.

[24] Abe S., Usami S.I., Shinkawa H., Kelley P.M., Kimberling W.J. (2000). Prevalent connexin 26 gene (GJB2) mutations in Japanese. J Med Genet.

[25] Chan D.K., Chang K.W. (2013). GJB2-associated hearing loss: systemic review of worldwide prevalence, genotype, and auditory phenotype. Laryngoscope.

[26] Erbe C.B., Harris K.C., Runge-Samuelson C.L., Flanary V.A., Wackym P.A. (2004). Connexin 26 and connexin 30 mutations in children with nonsyndromic hearing loss. Laryngoscope.

